# Application of 3-Dimensional Printing Technology to Construct an Eye Model for Fundus Viewing Study

**DOI:** 10.1371/journal.pone.0109373

**Published:** 2014-11-13

**Authors:** Ping Xie, Zizhong Hu, Xiaojun Zhang, Xinhua Li, Zhishan Gao, Dongqing Yuan, Qinghuai Liu

**Affiliations:** 1 Department of Ophthalmology, the First Affiliated Hospital of Nanjing Medical University, Nanjing 210029, P.R. China; 2 Department of Ophthalmology, the Second Affiliated Hospital of Nanjing Medical University, Nanjing 210000, P.R. China; 3 School of Optical Engineering, Nanjing University of Science and Technology, Nanjing 210094, P.R. China; 4 Ophthalmic Technology of College, Jinling Institute of Technology, Nanjing, 210001, P.R. China; University of Dayton, United States of America

## Abstract

**Objective:**

To construct a life-sized eye model using the three-dimensional (3D) printing technology for fundus viewing study of the viewing system.

**Methods:**

We devised our schematic model eye based on Navarro's eye and redesigned some parameters because of the change of the corneal material and the implantation of intraocular lenses (IOLs). Optical performance of our schematic model eye was compared with Navarro's schematic eye and other two reported physical model eyes using the ZEMAX optical design software. With computer aided design (CAD) software, we designed the 3D digital model of the main structure of the physical model eye, which was used for three-dimensional (3D) printing. Together with the main printed structure, polymethyl methacrylate(PMMA) aspherical cornea, variable iris, and IOLs were assembled to a physical eye model. Angle scale bars were glued from posterior to periphery of the retina. Then we fabricated other three physical models with different states of ammetropia. Optical parameters of these physical eye models were measured to verify the 3D printing accuracy.

**Results:**

In on-axis calculations, our schematic model eye possessed similar size of spot diagram compared with Navarro's and Bakaraju's model eye, much smaller than Arianpour's model eye. Moreover, the spherical aberration of our schematic eye was much less than other three model eyes. While in off- axis simulation, it possessed a bit higher coma and similar astigmatism, field curvature and distortion. The MTF curves showed that all the model eyes diminished in resolution with increasing field of view, and the diminished tendency of resolution of our physical eye model was similar to the Navarro's eye. The measured parameters of our eye models with different status of ametropia were in line with the theoretical value.

**Conclusions:**

The schematic eye model we designed can well simulate the optical performance of the human eye, and the fabricated physical one can be used as a tool in fundus range viewing research.

## Introduction

Since the Gullstrand's model eye was proposed in the early 20^th^ century [1], some new eye models have been devised by Navarro and Kooijman, which can better simulate the optical performance of the human eye [2]–[4]. Apart from these schematic eyes, other efforts had been made to fabricate concrete eye models that replicated both anatomical and optical properties of an average human eye. Those physical eye models were applied for visualization of IOL imaging properties [5], investigation of refractive errors [6], or assessment of the quality of soft contact lenses [7]. For vitreous-retinal surgeons, model eye also acts as a useful tool for assessing the fundus viewing during retinal surgeries like searching for peripheral holes and peeling epiretinal membrane. But the relevant reports have obtained their results only using optical ray-tracing methods [8]–[11], or evaluated the fundus visible location empirically instead of quantitatively [12], [13]. Until recently, based on Gullstrand's model, Inoue M constructed an eye model to evaluate the impact of multifocal, toric intraocular lens and magnifying prismatic lens on the fundus image viewing [14]–[16]. However, little information was provided in his paper to verify whether that model eye can correctly simulate the average human eye. Furthermore, it seemed too complicate to construct such a concrete eye model for wide application.

Three dimensional printing (3DP), firstly proposed by Hull C, is based on the common principal of building parts layer by layer [17]. Computer software splits the three dimensional computer aided design (CAD) or the CT(Computed tomography) data into a series of thin horizontal cross-sections (slices) and sends them to the printer machine for rapid prototyping sequentially from the bottom slice. Due to its accuracy, convenience and flexibility, 3D printing technology has been reported to be applied in many fields in medicine, such as the orthopedics, biomimetic structure, plastic surgery, and some tissue model for pre-surgical simulation [18]–[21]. However, there is no report about this technology in eye model construction until now.

The purpose of this study is to provide a method to simplify the procedure of constructing a physical eye model that can correctly simulate the optical performance of human eye. Based on the Navarro's eye (Table1) [4], we redesigned the parameters and compared the optical properties of our schematic model, Navarro's model eye and other two reported eye models[6], [7] on ZEMAX optical design software. With CAD software, we designed the 3D digital model and constructed our physical eye model. We also designed other three physical eye models with different statues of ametropia. To demonstrate the accuracy of the 3D printing, we measured the anterior chamber depth (ACD) and total axial length (TAL) and the results were compared with the theoretical ones. Furthermore, we assessed the fundus view range of the wide angle system to explore the future application of our physical eye model.

**Table 1:** Parameters of Navarro's model eye (relaxed) [4].

		Model eye 1(mm)	Model eye 2(mm)	Model eye 3(mm)	Model eye 4(mm)
ACD	Theoretical value	5	4	5	5
	UBM	4.990±0.0283	3.991±0.0093	4.988±0.0279	4.984±0.0136
	AS-OCT	4.977±0.0350	3.965±0.0345	4.983±0.0213	5.012±0.0299
	Lenstar	4.990±0.0337	3.983±0.0250	4.991±0.0260	4.983±0.0250
TAL	Theoretical value	23.86	23	24	25
	A-scan	23.852±0.0343	22.995±0.0143	23.969±0.0351	24.979±0.0443
	IOL Master	23.875±0.0558	22.981±0.0238	24.003±0.0125	24.998±0.0098
	Lenstar	23.865±0.0102	23.013±0.0210	23.998±0.0199	24.994±0.0241

ACD: anterior chamber depth; TAL: total axial length.

## Materials and Methods

### Instruments and materials

The cornea was made of polymethyl methacrylate (PMMA,n = 1.492(refractive index))(Orthok Technology Co., Ltd. Hefei. China)and the model body was printed with photosensitive resin by an Project 3510 HD printer(3D Systems Corporation, Rock Hill, USA). Iris was made by a piece of ring-shaped synthetic rubber and the diameter of pupil was changeable as needed. The Tecnis Z9003 (AMO, USA) IOL was chosen in this study. The balanced saline solution (BSS) was used to fill the anterior chamber and vitreous cavity because it had the similar refractive index with aqueous and vitreous humor.

The optical performance was analyzed using ZEMAX optical design software (Radiant Zemax, Redmond, WA) and the 3D digital model was designed on Solidwork 2008 software (Dassault Systemes S.A).

### Cornea

The calculation formulas of the corneal refractive power are described as the following: 
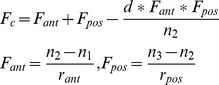
(1)


Where Fc refers to the total refractive power of the cornea, and the Fant and Fpos refer to the anterior and posterior power of the corneal surface, respectively; rant and rpos refer to the radius of the anterior and posterior surface respectively; d refers to the thickness of the cornea on the optical axial direction, and n1, n2, n3 refer to the refractive index of the air, cornea and aqueous humor, respectively. According to the parameters of the Navarro's eye and formula (1), the corneal power of Navarro's eye was +42.88 D and the effective focal length (EFFL) was 23.32 mm at the wavelength of 589.3 nm. The artificial cornea had the same central thickness and curvature of the anterior surface as that of Navarro's eye. The balanced saline solution we used had a refractive index of 1.3342 at wavelength of 589 nm and at 25°C. It was an average value of ten times measurements using WAY-2S Abbe refractometer (Jingke Industrial Co., Ltd. Shanghai. China). In order to keep the same power of the cornea of two model eyes, the radius of the posterior surface of the cornea in our model eye would be 7.41 mm.

Curved surface is commonly described by a conicoid equation:
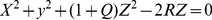
(2)


If Q equals to 0, the curved surface would be spherical, and if Q is between ⌜-1 and 0, the curved surface would be a prolate ellipse. Similar to the PMMA corneas of the reported model eye [14]–[16], [22], [23], the curve of the anterior surface of our cornea is prolate ellipse except possessing a spherical aberration of +0.27 um, which is comparable to the value of the average human cornea with a pupil diameter of 6 mm [24]. In ZEMAX, we set the spherical aberration(SPHA)to +0.27 um of the anterior surface of the PMMA cornea (surface1), which was the target value of the optimization, and the conic value (Q value) was set to be a variable factor. After default optimization at 0-degree, 5-degree, 10-degree, and 15-degree off-axial fields, Q equaled to −0.44 (pupil diameter  = 6 mm, chief wavelength = 0.5893 um) and it was the target asphericity for the fabrication.

### The position calculation and power calculation of the IOLs

SRK/T, one of the third era formulas for intraocular lens power calculation, contains (1) the postoperative anterior chamber depth (ACD, the distance from the corneal vertex to the central IOL) prediction, (2) corneal refractive index, and (3) retinal thickness correction factor [25]. We used SRK/T to calculate the ACD and IOLs power in our work. If the axial length of our schematic eye model was set to be 24 mm, based on the obtained PMMA corneal parameters, the ACD should be 5.0 mm and the power of the IOLs should be +19.1D for an emmetropic eye. However, the power of widely manufactured IOLs increases in steps of 0.50D in clinic, so we chose +19D Tecnis Z9003. Undoubtedly, this would bring defocus to the physical model eye. Then, focus optimization was performed using the auto-focus routine for minimum spot size radius by changing the vitreous cavity thickness in ZEMAX [26]. The ray tracing optimization yielded a total axial length (TAL) of 23.86 mm.

### Optical simulation on ZEMAX

Table 1 and Table 2 show the optical parameters of Navarro's eye and our schematic model eye. The parameters of the +19D Tecnis Z9003 IOLs were provided by AMO Company. The data of Arianpour et al.'s eye and Bakaraju's eye were obtained from their published paper [6], [7]. All ray-tracing simulations were performed at the condition of 589.3 nm wavelength and 3 mm-diameter pupil in ZEMAX. We got the spot diagram and spherical aberration in on-axis calculations. In off-axis calculations, following the methods of Arianpour [6], we quantified the seidel coefficients of the coma, astigmatism, field curvature, and distortion of four model eyes. We also compared the modulation transfer function (MTF) produced by the four model eyes because MTF curve represents the contrast transmission at various spatial frequencies of an optical system. Maximum MTF at 100 cycles/mm was chosen as the focusing criterion in accordance with the present ISO standard [27].

**Table 2:** Parameters of our schematic model eye.

no	notation	Radius r(mm)	Thickness d(mm)	Index (n)	Q value
1	Cornea	7.72	0.55	1.376	−0.26
2	Anterior chamber	6.5	3.05	1.3374	0
3	Crystalline lens	10.2	4.0	1.420	−3.1316
4	Vitreous humor	−6	16.3203	1.336	−1
5	Image	−12			

### Construction and measurements

Apart from the emmetropic model eye we have discussed above, other three physical eye models with different ACD (ranged from 4–5 mm) and TAL (ranged from 23–25 mm) were also printed and fabricated. We designed the 3D digital models of the two main parts of our physical eyes using those parameters discussed above by the Solidwork 2008 software. The digital model data was then input into the Project 3510 HD 3D printer for printing. An O-ring between the two parts of the model eye could prevent balanced saline solution leakage. PMMA cornea was made based on the parameters we got previously and a ring-shaped synthetic rubber cycling the cornea sealed the anterior chamber. The angle scale bars of vision field were set 90° at the equator and 0° at the fovea. A wavefront analyzer (Humphrey Atlas Corneal Topographer, Carl Zeiss Meditec Inc, Germany) was used to measure the spherical aberration of the anterior surface of the cornea. The anterior chamber depth was measured by ultrasound biomicroscopy (UBM, SW-3200L, Tianjin Suoer Electronic Technology Co., Ltd.), anterior segment optical coherence tomography (AS-OCT, Carl Zeiss Meditec International, Dublin, USA), and Lenstar LS900 (Hsgg-Streit International, Koeniz, Switzerland). Total axial length of the printed eye model was measured by A-scan (EchoScan US-1800; Nidek, Gamagori, Japan), IOL Master (Carl Zeiss, Jena, Germany), and Lenstar LS900 (Hsgg-Streit International, Koeniz, Switzerland). All of the parameters were measured 5 times for the average values.

The visible range of angle scale bars in the emmetropic physical model eye was analyzed under a wide-angle viewing system (Resight, Carl Zeiss Meditec AG, Jena, Germany) coupled with noncontact lens. The distance between the PMMA corneal surface and posterior surface of the 128-D or 60-D lens was set to 5 mm. The size of pupil was set to 6mm. The fundus pictures were photographed using a digital video camera attached to the surgical microscope.

## Results

**Figure 1:**
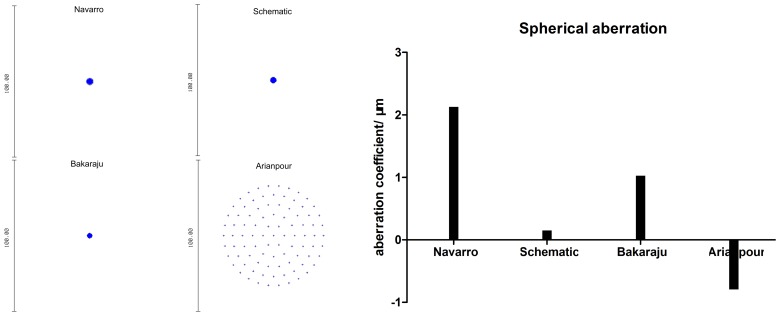
On-axis spot diagram and spherical aberration of four model eye in ZEMAX simulations. In ZEMAX, the object point is set at an infinite distance from the model eye, the wavelength is 589.3 nm and the pupil diameter is 3 mm.

**Figure 2:**
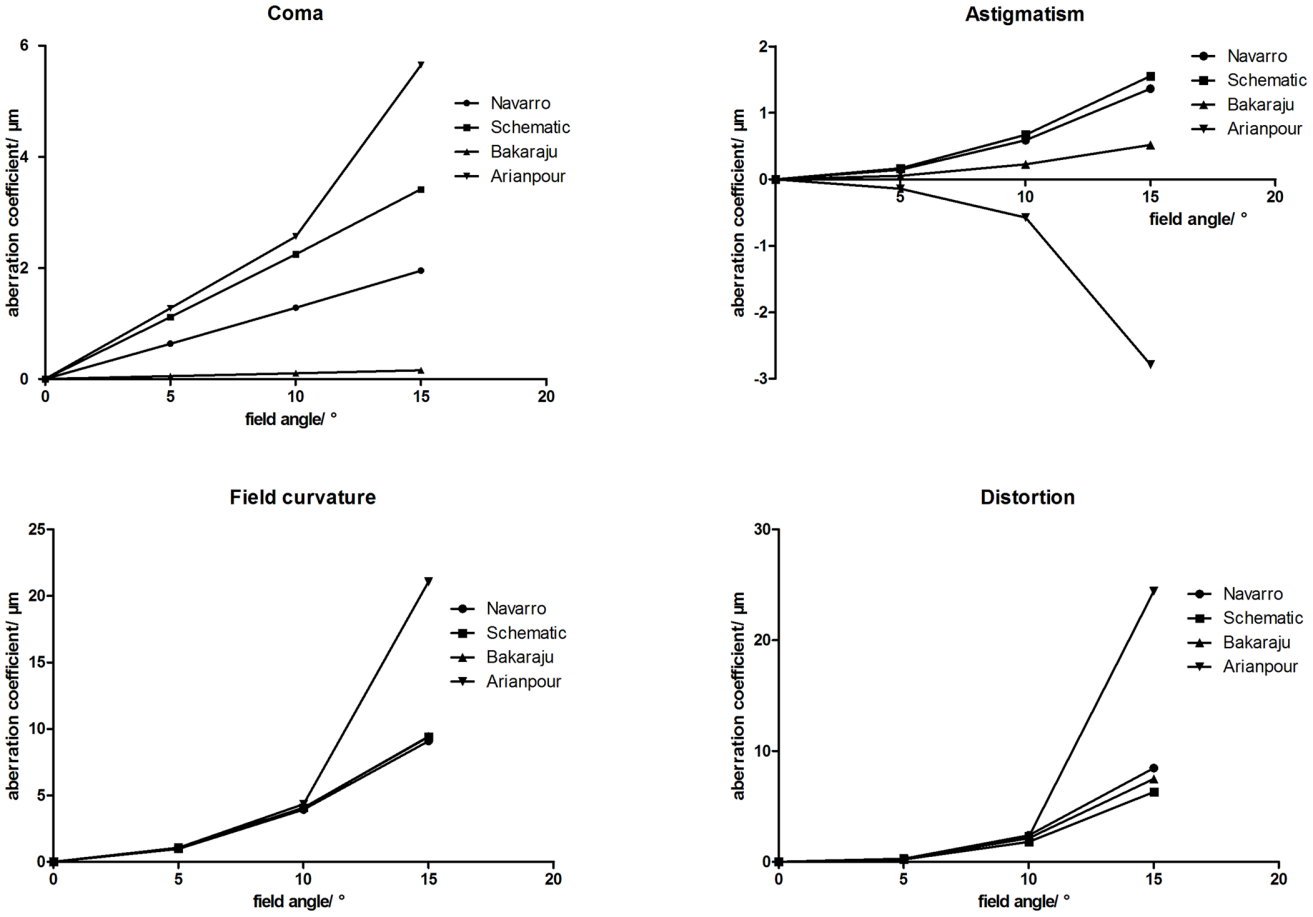
Aberrations of the four model eyes in ZEMAX. The seidel coefficients of the coma, astigmatism, field curvature, and distortion of four model eyes at 0-degree, 5-degree, 10-degree, and 15-degree off-axis simulation in ZEMAX software. The object point is set at an infinite distance from the model eye, the wavelength is 589.3 nm and the pupil diameter is 3 mm.

**Figure 3:**
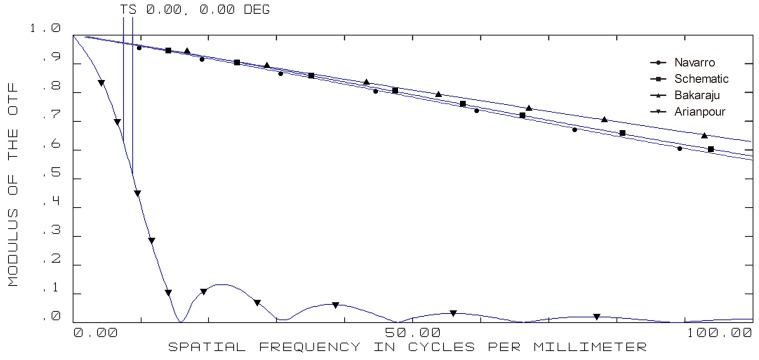
On-axis modulation transfer function (MTF) curves of four model eyes in ZEMAX. (wavelength: 589.3 nm; pupil diameter: 3 mm; T = tangential; S = sagittal).

**Figure 4:**
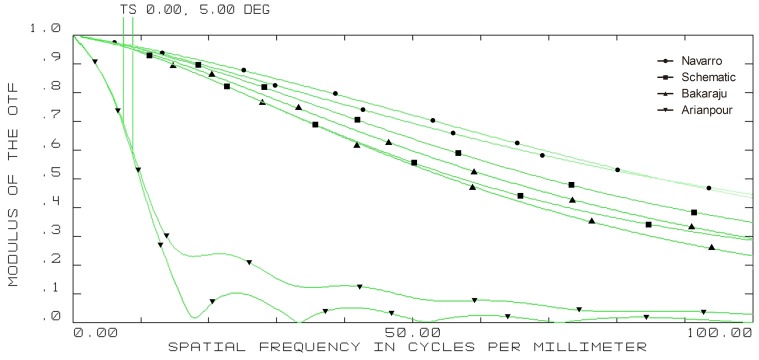
5-degree off-axis modulation transfer function (MTF) curves of four model eyes in ZEMAX. (wavelength: 589.3 nm; pupil diameter: 3 mm; T = tangential; S = sagittal).

**Figure 5:**
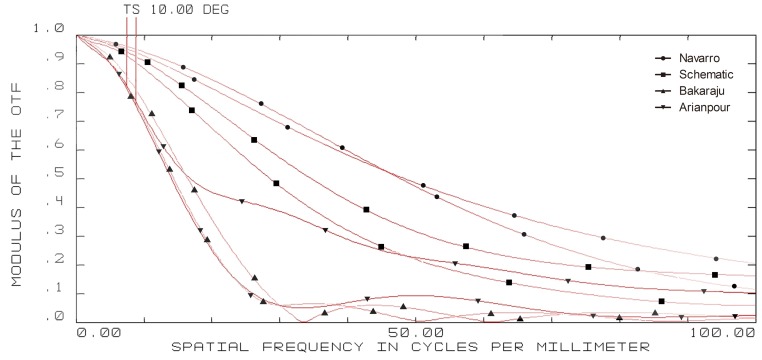
10-degree off-axis modulation transfer function (MTF) curves of four model eyes in ZEMAX. (wavelength: 589.3 nm; pupil diameter: 3 mm; T = tangential; S = sagittal).

The comparisons of the optical performance among the four model eyes were shown in Figure 1, 2, 3, 4, 5, and 6. Spot diagram (Figure 1A) refers to the pattern of rays as they focus on the retina referenced to the chief ray. The RMS(root-mean-square)radius of the spot diagram gives an average level of all the radiuses of the spreading rays. The RMS radius of our schematic eye (1.256 µm) is smaller than Navarro's (1.342 µm) and larger than Bakaraju's (0.771 µm). But the RMS radius of the spot diagram of Arianpour's eye was much larger (25.78 µm). Our schematic model eye possesses the lowest spherical aberration, the coefficient was 0.15 µm, while it is 2.172 µm, 1.025 µm, and −0.794 µm for Navarro's, Bakaraju's and Arianpour's eyes respectively (Figure 1B).

**Figure 6:**
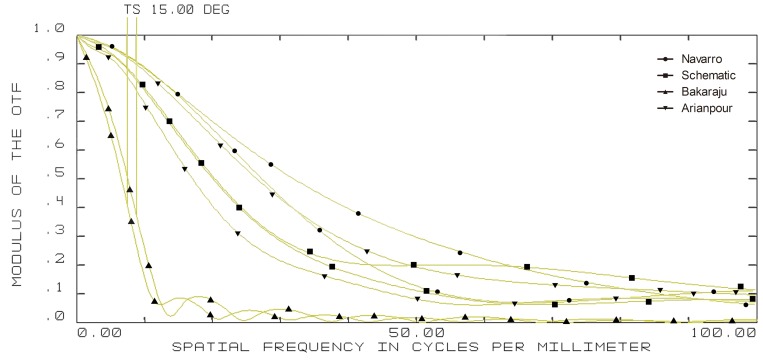
15-degree off-axis modulation transfer function (MTF) curves of four model eyes in ZEMAX. (wavelength: 589.3 nm; pupil diameter: 3 mm; T = tangential; S = sagittal).

The changes of the seidel aberration coefficients of the off-axial optical quality— coma, astigmatism, field curvature and distortion were shown in Figure 2. We can see from Figure 2, the coefficients of seidel aberration all increase with the field angle enlarging. The Arianpour's eye has the largest coma coefficient at 15-degree (5.651 µm), followed by our schematic eye (3.471 µm), Navarro's eye (1.952 µm), and Bakaraju's eye(0.160 µm). Astigmatism coefficient at 15-degree of our schematic eye model was 1.553, followed by Navarro's eye (1.364 µm) and Bakaraju's eye (0.518 µm). While it is a negative value in Arianpour's eye (-2.785 µm). As for the field curvature, it is nearly identical among Navarro's eye (9.083 µm), our schematic eye (9.405 µm), and Bakaraju's eye (9.433 µm), while it is much higher in Arianpour's eye (21.066 µm). The distortion coefficients at 15-degree of our schematic eye, Navarro's eye, and Bakaraju's eye are 6.307 µm, 8.463 µm, and 7.496 µm respectively. But Arianpour's eye has a much higher distortion, which is 24.414 µm.

The on-axis and off-axis MTF was also calculated for all model eyes (Figure 3, 4, 5, and 6). Our schematic eye is similar to Navarro's eye at all the four field angles. Both MTF curves of the above two model eyes diminish a bit more quickly than that of Bakaraju's eye on axis (Figure 3), but much more slowly than Bakaraju's eye and Aranpour's eye at 5-degree (Figure 4), 10-degree (Figure 5), and 15-degree (Figure 6) off-axis fields. At 10-degree and 15-degree, the MTF of Bakaraju's eye drops the most quickly among the four model eyes (Figure 5, 6).

**Figure 7:**
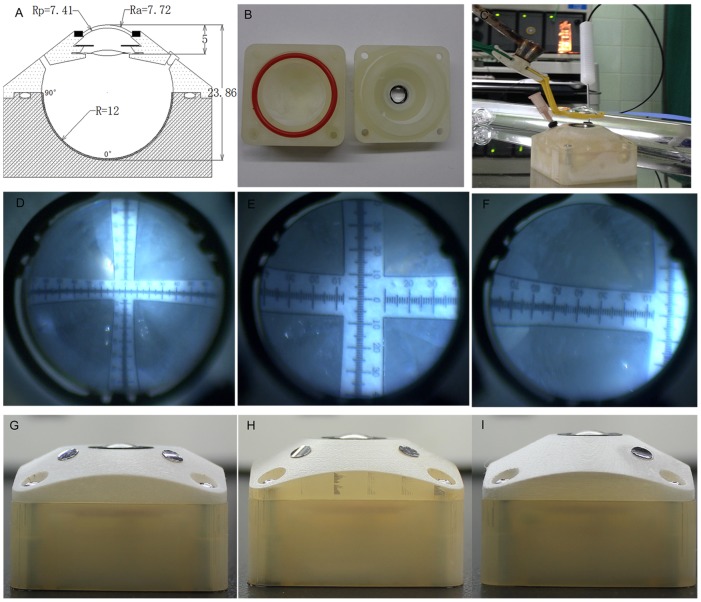
Fabrication and use of the physical eye model. A: Schematic view of the cross-section of our physical model eye; B: two printed parts provided main structure of the physical model eye; C: use of the physical eye model for assessing the fundus range of the viewing system; D–F: pictures of the angle bars photographed under 128D lens, 60D lens, and 60D lens with model eye tilt; G–I: other three eye models printed and fabricated with different anterior chamber and total axial length.

In our physical eye model the 128D lens provided an approximate 70° (Figure 7D) while the 60D lens provided an approximate 40°fundus range (Figure 7E).The bars under 60D lens seemed clearer than that under 128D lens. We also founded that the fundus range could extend to 79° when the physical model eye was tilted to 40° (Figure 7F). Other three physical eye models with different refractive statues were successfully printed and fabricated (Figure 7G–7I).

The mean spherical aberration of the anterior surface of the physical model eye was +0.268 µm for a 6 millimeter diameter pupil. The measured results of ACD and TAL were shown in Table 3.

**Table 3:** Measurements of our fabricated eye models.

no	notation	Radius r(mm)	Thickness d(mm)	Index (n)	Q value
1	Cornea	7.72	0.55	1.492	−0.44
2	Anterior chamber	7.41	3.994	1.3342	0
3	Tecnis Z9000	12.7173	0.912	1.47	−1.30613
4	Vitreous humor	−15.7226	18.405	1.3342	0
5	Image	−12			

## Discussion

Model eyes can be used to simulate the optical performance of normal and pathologic eyes, and to develop and evaluate optical corrections designed to improve retinal image quality. Different from schematic model eyes, physical model eyes offer the best alternative to address the shortcomings of them. Based on their utility, physical eyes can be applied to calibrate ophthalmic instruments, for education and training, and most optically relevant, for evaluating the characteristics of refractive elements, such as IOL, soft contact lens [7]. Therefore, building a simple and low cost approach to construct a physical eye model which can simulate the optical properties of human eye will be meaningful in ophthalmological researches. In this study, we have designed the optical parameters of our model eye based on Navarro's eye, compared its on-axis and off-axis optical property with reported physical eyes, and used the 3D printing technology to construct it for the first time. The life-sized physical eye we fabricated is available for the assessment of visible fundus range under various viewing conditions.

### The parameters of our model eye

Our schematic model eye was designed based on Navarro's eye with some changes. The refractive index of the balanced saline solution filled the model eye in Eppig T's research was assumed to be 1.336 [28], while in Gobbi's study, it was 1.334 [5]. Considering the difference among the publications, and it affects the PMMA cornea power calculations (Formula 1), we measured it using Abbe refractometer. When the cornea of our concrete eye model was made of PMMA(n = 1.492), we changed the radius of the posterior surface of the cornea from 6.5 mm (Navarro's eye) to 7.41 mm(our new model eye) to keep the same refractive power as that of Navarro's cornea (+42.88 D). This was similar to Inoue M′ design, which changed the radius of the posterior surface of the cornea from 6.8 mm(Gullstrand's eye) to 7.46 mm to keep the same refractive power as Gullstrand's cornea [14]–[16]. The ACD, IOLs power and the TAL were obtained from SRK/T formulas and ZEMAX calculations. Then optical performance of our schematic eye was then compared with other model eyes in ZEMAX optical design software.

### Optical simulation of the model eye

We chose to compare the optical properties of our schematic eye with Bakaraju's eye and Arianpour's eye based on two reasons. On one hand, these two model eyes were recently fabricated and provided sufficient optical parameters. On the other hand, same as ours, both model eyes were designed based on the widely-accepted Navarro's eye. Although the parameters of Navarro's eye were defined at the wavelength of 589.3 nm [4], the simulations in software of Bakaraju's and Arianpour's model eye were performed at reference wavelength of 580 nm [7] and 550 nm [6] respectively. The reason we choose 589.3 nm in ZEMAX is that refractive indexes of our physical eye were all obtained at 589.3 nm and the wavelength in Bakaraju's study was also close to 589.3 nm.

Ray-tracing simulations in ZEMAX suggested the size of spot radius were very close among Navarro's, Bakaraju's, and our schematic model eye while it was large in Arianpour's eye. The results also indicated that our schematic eye focused well while there's defocus in Arianpour's eye. The coefficient of spherical aberration of our schematic eye was the smallest among the four models. For other items of analyzing the off-axial optical quality, our schematic eye performed the closest to Navarro's eye (Figure 2). The MTF curves (Figure 3, 4, 5, and 6) implied that Arianpour's eye performed worse than the other three model eyes, Bakaraju's eye performed well on axis while worse with the increase of the field of view, and our schematic model eye performed pretty similar to that of Navarro's eye. The reason why the optical performance of Arianpour's eye in ZEMAX was not that perfect might lied in 1), Arianpour designed his model eye based on the firstly-proposed Navarro's eye in 1985[3] instead of the latter one in 1999 (also called Navarro-Escudero's model eye)[4]. 2), Its parameters were obtained from calculations at the wavelength of 550 nm instead of 589.3 nm. Bakaraju has founded the chromatic shifts in refraction (D) relative to different reference wavelengths [7] and it also been demonstrated in our ZEMAX simulations (data were not shown).

### Characteristics of the 3D printed model eye

The construction of the traditional eye models demanded more complex parts of molds [6], [7], [14]–[16] while in our study, only two digital models were designed on CAD software for quickly printing. The 3D printer can be classified into ordinary level and industrial level in a rough way. We used the industrial level printer to fabricate the eye model and the high resolution of the printer reaches 20 µm. To test the accuracy of the 3-D printing process and other aspects of the manufacturing process, we have fabricated four physical model eyes with different ACD and TAL to simulate different states of ametropia and their parameters were measured by several kinds of ophthalmological instruments. The parameters of the measurements were in line with what we designed, which manifested the high resolution and accuracy of the Projet HD 3D printer [29].

Another feature of the 3D printing technology is it supplies us the flexibility of revision during the whole procedure. Furthermore, besides the emmetropic condition, we can also apply this technology to obtain the eye models with different statues of ametropia. By changing the radius of the PMMA cornea and the total axis length the physical model eye, we could fabricate eyes models of refractive ammetropia and axial ammetropia respectively.

The printed eye model in our research will supplied a useful tool for further fundus viewing studies. In this paper, we evaluated the visible fundus range of 128D and 60D noncontact lens in the wide angle viewing system. In future, we can further quantitatively measure the image under various viewing conditions (e.g. different fundus viewing devices, different kinds of IOLs, and different statues of ammetropia).

However, there are still a few limitations associated with our physical eye model. One is that when different IOLs were implanted to simulate pseudophakic eyes, total axial length must be calculated in ZEMAX for good focus before further fabrication. The other is that the physical eye model has not been explored to capture visual image in this paper. A complementary metal-oxide semiconductor (CMOS) [6] or charge coupled device (CCD) [28] camera might be helpful to record the visual image in future work.
